# Civic engagement and psychological distress during the COVID-19 pandemic

**DOI:** 10.1186/s12889-022-13289-4

**Published:** 2022-05-02

**Authors:** Rachel J. Topazian, Adam S. Levine, Emma E. McGinty, Colleen L. Barry, Hahrie Han

**Affiliations:** 1 Department of Health Policy and Management, Johns Hopkins Bloomberg School of Public Health, 624 N. Broadway, Baltimore, MD 21205 USA; 2 Cornell Jeb E. Brooks School of Public Policy, Ithaca, NY USA; 3 Stavros Niarchos Foundation Agora Institute, Johns Hopkins University, Baltimore, MD USA

**Keywords:** Civic associations, Political engagement, Psychological distress, Interpersonal interactions, COVID-19 pandemic

## Abstract

**Background:**

To examine the relationship between civic association participation and psychological distress during the COVID-19 pandemic, particularly whether different forms of engagement mitigate the increased rates of psychological distress throughout 2020.

**Methods:**

Panel survey data collected from a nationally representative cohort of 1222 U.S. adults. Data was collected in three waves in April, July, and November 2020. Psychological distress was measured using the validated Kessler-6 instrument in November 2020.

**Results:**

Respondents belonging to political associations were more likely to experience psychological distress (difference in predicted level of psychological distress on a 0-1 scale: 0.098, *p* ≤ .05) relative to those in unknown associations. However, individuals in political associations who more frequently interacted with others had lower levels of psychological distress (−.065, *p* ≤ .05) compared to those in political associations with less frequent interactions.

**Conclusions:**

Civic engagement that facilitates interpersonal interactions may protect against psychological distress.

**Supplementary Information:**

The online version contains supplementary material available at 10.1186/s12889-022-13289-4.

## Background

The COVID-19 pandemic prompted serious mental health concerns, due to increased social isolation and concurrent crises, including the widespread public debate about systemic racial inequality in the United States and the general social disaffection caused by a highly polarized 2020 presidential election [1, 2]. Research shows sharply elevated rates of psychological distress throughout 2020 compared to previous years. The rate of psychological distress in the U.S. adult population was approximately 4% in 2018, but jumped to 13.6% in April 2020 and remained high (13.0%) in July 2020 [3]. This is likely due in part to social distancing guidelines and the resulting closure of businesses, schools, and places of worship, which increased social isolation. It may also reflect the general sense of social upheaval and uncertainty caused by the pandemic and by the tumult over politics, racial inequality, and other polarized debates throughout 2020. Prior research suggests that U.S. elections are a source of stress in the general population and that greater political engagement is associated with reporting of negative psychological outcomes [4, 5].

It is possible that the social connectedness that emerges from involvement with civic associations could ameliorate the social isolation that exacerbated mental health issues in 2020 [6]. Civic engagement includes a broad array of activities ranging from association membership, to involvement in local community organizations, to volunteering, to explicitly political activities like voting or working on a campaign [7]. Civic engagement, broadly defined, has been linked to better physical and mental health outcomes [8, 9], and there is substantial evidence that volunteering with civic associations is associated with lower levels of depression and psychological distress [10, 11]. This research suggests that civic associations may have had positive effects on mental health during the pandemic, as they may have kept people engaged with their communities and each other in virtual ways. However, we are unaware of studies assessing the relationship between different forms of civic association affiliations and psychological distress during the COVID-19 pandemic.

Drawing on original panel data and novel measures of civic association affiliations, this paper examines the relationship between civic engagement and prevalence of psychological distress during the COVID-19 pandemic. The nature of engagement could have varying effects on mental health, as the amount of time committed to an association and frequency of personal interactions with association members could mitigate the negative effects of social isolation. The impacts of civic association affiliations may also have differed for individuals in political associations (defined as groups that explicitly make claims on government, unlike civic associations, such as hobby clubs, that do not) relative to other forms of civic engagement, as the pandemic coincided with a highly contentious election cycle. Based on these considerations, we hypothesized that spending greater amounts of time with an association and more frequently interacting with people at an association would have protective effects on psychological distress, and that affiliation with political associations would have adverse impacts.

## Methods

The *Johns Hopkins COVID-19 Civic Life and Public Health Survey* is a longitudinal, nationally representative panel survey fielded online in three waves in 2020: April 7-13, 2020 (wave 1), July 7-22, 2020 (wave 2), and November 11-30, 2020 (wave 3). We used the NORC AmeriSpeak® panel, which is a probability-based panel designed to be representative of the U.S. adult population [12]. The panel covers 97% of U.S. households and is sampled from U.S. Postal Service addresses and a NORC area probability sample. Participants are recruited using mailings, telephone calls, and in person visits. The Amerispeak panel has a response rate of approximately 34%. Additional file 1: Appendix A compares the study population to national statistics. There were 1468 Wave 1 respondents (70.4% completion rate), 1337 Wave 2 respondents (91% completion rate), and 1222 Wave 3 respondents (92% completion rate). Participants received small cash incentives. This study was approved by the Johns Hopkins Bloomberg School of Public Health Institutional Review Board.

### Measures

We collected data on our outcomes of interest in November 2020, in the weeks following the U.S. presidential election. Additional file 1: Appendix B contains exact wording for all survey questions. We asked participants about their psychological distress using the Kessler-6 Psychological Distress Scale, a validated 24-point scale evaluating psychological distress [13]. The Kessler-6 consists of six items that ask how frequently respondents experienced psychological distress symptoms in the last 30 days. Each item is measured using a 5-point Likert scale ranging from none of the time (0) to all of the time (5). Responses from all six questions are summed to produce a final score that can range from 0 (low distress) to 24 (severe distress). To increase interpretability, we rescaled this outcome on a 0-1 scale. Coefficients reflect differences in psychological distress on a 0-1 scale in which 0 represents low psychological distress and 1 represents severe psychological distress.

In Wave 1, we gave participants a list of 16 well-established categories of civic associations [14], and asked them to identify the number of associations within each category that they affiliate with. The first step of this prompt is to help respondents recall the range of different types of associations they might interact with. We then asked them to name the association most important to them. Participants who did not name an association, or whose response from Wave 1 was unclear, were asked this question again in Wave 2. We then categorized each association into seven types: Business or Professional, Community, Arts and Recreation, Identity-Based, Political, Religious, Social Services, and Unknown. Two team members checked categorizations of the association most important to each respondent. Respondents who did not name their most important association or whose association names could not be identified were categorized as Unknown.

In Wave 1, we asked participants to describe their relationship with the association most important to them. We asked respondents to estimate the number of hours they devoted to the association each week (e.g., reading emails or attending events) using a five-category scale (none to more than 10 hours). We also asked how often the respondent interacted with people in the association (never, occasionally, regularly).

In Wave 3 (November 2020) we measured covariates including self-reported health status, using a five-point scale ranging from poor to excellent. We also asked participants to self-report if they were essential workers during the pandemic using a dichotomous response. We utilized additional variables from the NORC baseline panel data, including: gender (male, female), race (White, Black, Hispanic, Asian/Other), age, household income (18-point scale), education level (14-point scale), and marital or partnered status.

### Statistical analysis

We conducted multivariate linear regression to estimate predictors of psychological distress, developing four models to examine the main effects of: 1) hours spent with the association and type of association, 2) frequency of interactions with people in the association and type of association, 3) the interaction between hours spent with the association and belonging to a political association, and 4) the interaction between frequency of interactions with people and belonging to a political association. In each model we controlled for gender, race/ethnicity, age, income, education, essential worker status, and married/partnered status. We used one-tailed significance tests for the interactions between belonging to a political association and hours spent with the association and frequency of interpersonal interactions, based on the directional nature of our hypothesis that greater engagement would be associated with lower psychological distress. We conducted a sensitivity analysis measuring moderate psychological distress using dichotomized versions of each variable (Additional file 1: Appendix C). Analysis was conducted using survey weights to calculate nationally representative estimates in Stata Version 16 [15].

## Results

Of the 1222 respondents who completed all three waves, 83.2% identified an association. When asked to name the association most important to them, 5.4% named a business or professional association (e.g., National Association of Realtors, Kentucky Educators Association); 12.0% named a community, arts, or recreation association (e.g., Portland Art Museum, YMCA); 17.2% named an identity-based association (e.g., American Legion, The Junior League); 6.2% named a political association (e.g., Biden Campaign, National Rifle Association); 22.3% named a religious association (e.g., Catholic Church, Lutheran Church Charities); 13.9% named a social services association (e.g., Feeding America, St. Jude Research Hospital); and 6.2% named an unknown type of association.

15.1% of respondents indicated that, on average, they spent no hours interacting with their association each week; 48.0% spent 1-2 hours per week; 21.4% spent 2-5 hours per week; 10.2% spent 5-10 hours per week; and 5.2% spent more than 10 hours with their association each week. 16.6% reported never interacting with people in the association; 49.6% reported occasionally interacting with people; and 33.8% indicated they regularly interacted with people in the association.

Among the different types of associations, individuals whose primary affiliation was a political association were more likely to experience psychological distress (0.098, *p* ≤ .05). This main effect was consistent across models controlling for hours spent interacting with the association each week and frequency of interactions with people at the association (Table 1). We did not observe statistically significant differences in psychological distress for individuals in political associations based on their hours spent with the association. Respondents who had more frequent interactions with people in a political association reported statistically significant lower levels of psychological distress (−.065, *p* ≤ .05).

**Table 1:** Effects of civic engagement on psychological distress among U.S. adults in November 2020

	Model 1^a^	Model 2^b^	Model 3^c^	Model 4^d^
	*n* = 1000	*n* = 999	*n* = 1000	*n* = 999
**Type of Organization**	Linear regression coefficient (standard error)			
Unknown	ref	ref	ref	ref
Business or Professional Organization	−0.010 (0.050)	− 0.004 (0.049)	− 0.010 (0.050)	− 0.004 (0.049)
Community, Arts & Rec Organizations	0.033 (0.044)	0.045 (0.045)	0.033 (0.044)	0.042 (0.045)
Identity-based Organizations	0.046 (0.043)	0.053 (0.042)	0.046 (0.043)	0.051 (0.042)
Political Organizations	0.098* (0.044)	0.102* (0.043)	0.089 (0.081)	0.222** (0.081)
Religious Organization	−0.029 (0.040)	−0.012 (0.039)	− 0.029 (0.040)	− 0.018 (0.040)
Social Services Organizations	0.016 (0.048)	0.024 (0.047)	0.016 (0.048)	0.021 (0.048)
**Type of Engagement/ Nature of interactions**				
Average hours interacting with the organization each week	0.008 (0.009)		0.008 (0.009)	
Frequency of interactions with people at the organization		−0.011 (0.014)		−0.005 (0.015)
Political org x hours per week			0.004 (0.035)	
Political org x frequency of interactions with people				−0.065*^1^ (0.036)
**Demographic Controls**				
Female	0.046* (0.019)	0.048* (0.019)	0.046* (0.019)	0.048* (0.019)
Black, non-Hispanic	−0.059* (0.027)	−0.058* (0.027)	− 0.059* (0.027)	−0.057* (0.027)
Hispanic	−0.030 (0.034)	−0.031 (0.034)	− 0.030 (0.034)	−0.030 (0.034)
Asian, Other	−0.044 (0.061)	−0.042 (0.061)	− 0.044 (0.061)	−0.041 (0.061)
Age	−0.004*** (0.001)	−0.004*** (0.001)	− 0.004*** (0.001)	−0.004*** (0.001)
Household Income	−0.001 (0.003)	−0.001 (0.003)	− 0.001 (0.003)	−0.001 (0.003)
Education Level	−0.006 (0.007)	−0.005 (0.007)	− 0.006 (0.007)	−0.006 (0.007)
Essential Worker	−0.036 (0.023)	−0.036 (0.023)	− 0.035 (0.023)	−0.037 (0.023)
Married/Partnered	−0.060* (0.025)	−0.058* (0.025)	− 0.060* (0.025)	−0.060* (0.025)
**Constant**	0.545*** (0.090)	0.563*** (0.087)	0.545*** (0.090)	0.553*** (0.087)

*p ≤ 0.05, **p ≤ 0.01, ***p ≤ 0.001 statistically significant from reference category. Standard errors are in parentheses. Psychological distress was measured using the Kessler-6 scale, rescaled from 0 to 1, with 0 representing low distress and 1 representing severe distress. Associational characteristics were collected in Wave 1. Sex, race/ethnicity, age, household income, education, and married/partnered status were collected as part of the baseline NORC Amerispeak panel. Psychological distress and the essential worker variable were evaluated in Wave 3

^1^ Indicates use of a one-tailed significance test, based on our hypothesis that more hours spent with an association and more interpersonal interactions would be associated with lower rates of psychological distress

^a^Model 1 examines the main effects of hours spent with the association and type of association

^b^Model 2 examines the main effects of frequency of interactions with people in the association and type of association

^c^Model 3 examines the main effects of the interaction between hours spent with the association and belonging to a political association

^d^Model 4 examines the main effects of the interaction between frequency of interactions with people and belonging to a political association

## Discussion

Our findings reveal important links between a person’s civic engagement and their level of psychological distress during the COVID-19 pandemic. Consistent with our expectation that the political events of 2020 elevated psychological distress, our findings reflect higher psychological distress among individuals who consider political associations most important to them, indicating that this form of civic engagement might have complex effects on wellbeing. This likely reflects the timing of our data collection in the weeks following the November 2020 elections, which were characterized by intense political polarization and misinformation [16, 17]. The contentious presidential election cycle likely contributed to increased psychological distress for individuals affiliated with political groups relative to their less politically-engaged peers. Political activities such as working on a campaign or engaging with public officials are important avenues of civic engagement that have been encouraged both as intrinsically valuable activities and for their associations with improved mental health [7, 9]. Given the increasingly polarized political climate in the U.S., it will be important to track and address elevated levels of psychological distress among individuals actively engaging with political groups.

We also discovered that the nature of an individual’s interactions with an association affected the relationship between civic engagement and psychological distress. In general, simply spending more hours with a political association did not alter psychological distress. However, respondents in political associations who had more frequent interactions with people reported reduced psychological distress compared to those with less frequent interactions. Interpersonal interactions might be more important for subgroups experiencing higher rates of psychological distress. This might also imply that the nature of engagement, and personal interactions in particular, are important factors determining the relationships between civic engagement and mental health. Our findings may also reflect the importance of interpersonal interactions during the pandemic, when many Americans were limiting social interactions [6]. We assessed levels of psychological distress in November 2020, when many states were limiting large gatherings or imposing stay at home advisories [18]. Future research should examine how different forms and intensities of interpersonal engagement might affect psychological distress beyond the COVID-19 pandemic.

Our study has several limitations. While the AmeriSpeak® panel uses probability-based recruitment aligning with best-practices, our results might be affected by sampling bias. We have limited ability to detect statistically significant differences among subgroups with small sample sizes. While two members of our team classified each association, there were a small number (6.2%) that we were unable to categorize. Our estimate of affiliations with an association (83%) is higher than recent survey estimates that ask about participation in a community organization (57%) or census estimates of formal organization membership (26%) [19, 20]. This may reflect our inquiries about any affiliation; a more stringent definition of civic engagement might produce different results. Our measures of respondents’ interactions with their associations are self-reported and may be subject to recall bias. We lack comparisons for our civic association variables pre-pandemic, and cannot evaluate how interactions may have changed during the pandemic. Our results are also restricted to the United States and may not be generalizable to other settings. Finally, we lack data on respondents’ social distancing behaviors and are unable to determine how their interactions with civic associations and level of interpersonal interactions in general changed during the pandemic. More research is needed to examine the effects of civic association engagement as the pandemic wanes.

## Conclusion

The pandemic’s threats to mental health will likely endure beyond its physical health impacts, prompting the need for long-term strategies to bolster mental health outcomes. Our work introduces new measures of civic association involvement and sheds light on the protective effects of interpersonal interactions in associations on psychological distress, offering new tools for public health leaders to utilize in pandemic recovery.

## Supplementary Information

**Additional file 1.**

## Data Availability

Data is not available. Supplementary material is included in the Appendix.
