# Supplementary material for: Civic engagement and psychological distress during the COVID-19 pandemic
Source: BMC Public Health. 2022 May 2;22:869. doi: 10.1186/s12889-022-13289-4 (PMC9058736; doi:10.1186/s12889-022-13289-4)
Supplement: Supplementary file 1 — Additional file 1. [file 12889_2022_13289_MOESM1_ESM.docx]

**Appendix**

**A. Unweighted and Weighted Characteristics of Respondents Compared to the U.S. Population**

|  | | National Comparison of Adults (Age 18+) | Wave 1 Unweighted | Wave 1 Weighted | Wave 3 Unweighted | Wave 3 Weighted |
| --- | --- | --- | --- | --- | --- | --- |
|  |  |  | (n=1,468) | (n=1,468) | (n=1,222) | (n=1,222) |
| Female (%) | | 51.3 | 51.2 | 51.7 | 51.1 | 52.1 |
| Age (%) | |  |  |  |  |  |
| 18-34 | | 29.7 | 28.7 | 31.7 | 21.5 | 27.7 |
| 35-49 | | 24.5 | 22.6 | 21.9 | 25.9 | 22.6 |
| 50-64 | | 24.6 | 26.8 | 24.9 | 27.4 | 26.3 |
| 65+ | | 21.2 | 22.0 | 21.5 | 25.1 | 23.4 |
| Race/Ethnicity (%) | |  |  |  |  |  |
| White only | | 62.8 | 66.1 | 62.8 | 69.0 | 65.5 |
| Black only | | 12.1 | 13.8 | 11.9 | 12.9 | 11.2 |
| Other | | 8.7 | 5.8 | 8.6 | 4.9 | 7.7 |
| Hispanic | | 16.4 | 14.3 | 16.7 | 13.2 | 15.6 |
| Education (%) | |  |  |  |  |  |
| High school diploma or less | | 52.5 | 19.6 | 38.0 | 18.4 | 36.9 |
| Some college or higher | | 47.5 | 80.4 | 62.0 | 81.6 | 63.1 |
| Household income (%) | |  |  |  |  |  |
| <$35,000 | | 19.5 | 29.1 | 31.5 | 27.3 | 29.0 |
| $35,000 | | 28.2 | 34.6 | 32.5 | 35.4 | 33.0 |
| -$74,999 | | 52.3 | 36.3 | 36.0 | 37.4 | 38.0 |
| Political Party Affiliation (%) | |  |  |  |  |  |
| Republican | | 29.5 | 26.2 | 25.9 | 26.7 | 27.2 |
| Independent | | 33.8 | 39.7 | 41.7 | 39.2 | 39.7 |
| Democrat | | 36.7 | 34.1 | 32.4 | 34.2 | 33.0 |

National comparison data were obtained from the 2019 Current Population Survey (CPS) and from the 2016 American National Election Studies (ANES): [http://www.electionstudies.org/studypages/anes_timeseries_2016/anes_timeseries_2016.htm](about:blank).

Percentages from the survey are calculated excluding missing values.

In this table, we apply the survey weight that corresponds to the survey wave.

**B. Survey Question Wording**

1. Some people like belonging to civic and political organizations that bring people together around a common issue, cause, identity group, or other common interest. Other people do not like being part of these organizations. Think about *all* the groups you are involved with at any level, including groups where you simply receive emails, donate money, etc. *however minimal your involvement is.* Include groups in which you are actively and passively involved—include any organizations with which you interact or receive information from, even if you are not a formal member. For each type of organization below, please indicate how many organizations you affiliate with in this category.
2. Service and Fraternal organizations
3. Veterans’ organizations
4. Religious organizations
5. Senior citizen organizations
6. Women’s organizations
7. Organizations defined by nationality, ethnic or racial identities
8. Labor unions
9. Business or professional associations
10. Issue-based political advocacy organizations
11. Electoral organizations
12. Youth organizations
13. Literary, arts, and cultural organizations
14. Recreational and sports organizations
15. Neighborhood groups
16. Health or social services organizations
17. Educational organizations
18. Of these, please name which organization is most important to you? [open ended response]

Respondents who did not provide an answer to question 2 in Wave 1 or who responded “None” were asked question 2 again in Wave 2. Only respondents who did not provide an answer to question 2 in Wave 1 or responded “None” received question 3.

1. When we asked you in April for the name of the organization that is most important to you, you didn’t name one. Are you sure that none of these organizations is important to you?
   1. Yes, I am sure. I do not want to name any of these types of organizations as important to me. [If YES, SKIP REST OF QUESTIONS IN MODULE]
   2. The organization that is most important to me is ____________________ [please spell out any acronyms]

Respondents whose responses to question 2 in Wave 1 were not clear were prompted to answer question 4.

1. When we asked you in April for the name of the organization that is most important to you, your answer was not clear to us. Can you please respond again.
   1. The organization that is most important to me is ____________________ [please spell out any acronyms]
2. If you average across the past twelve months, about how many hours per week did you devote to the activities of this organization, including reading emails or letters they send you, attending events, or interacting in other ways?
   1. None
   2. 1-2 hrs/week
   3. 2-3 hrs/week
   4. 5-10 hrs/week
   5. More than 10 hrs/week
3. Now we want to ask you about the kinds of interactions you have with people in the organization. When we ask you about these interactions, they can be online or offline, with staff or volunteers of the organization, or with other people who are members of or affiliate with the organization. First, how often do you interact with people in the organization?
   1. Never
   2. Occasionally
   3. Regularly

Respondents were asked to respond to question 7 using a five point scale.

| All of the time | Most of the time | Some of the time | A little of the time | None of the time |
| --- | --- | --- | --- | --- |

1. During the past 30 days, about how often did you feel…
   1. Nervous?
   2. Hopeless?
   3. Restless or fidgety?
   4. So depressed that nothing could cheer you up?
   5. That everything was an effort?
   6. Worthless?
2. Did the COVID-19 pandemic affect your job in any of the following ways? Select all that apply.
   1. My job was deemed “essential” during the COVID-19 pandemic.

***Respondents were asked to answer questions 9 through 17 as part of their enrollment in NORC’s AmeriSpeak Panel.***

1. Are you
   1. Married
   2. Widowed
   3. Divorced
   4. Separated
   5. Never married
   6. Living with partner

For questions 10 through 17, respondents were given the following prompt: The next question is about the total income of YOUR HOUSEHOLD for [INSERT LAST YEAR EG: 2014 IF TODAY IS 2015]. Please include your own income PLUS the income of all members living in your household (including cohabiting partners and armed forces members living at home). Please count income BEFORE TAXES and from all sources (such as wages, salaries, tips, net income from a business, interest, dividends, child support, alimony, and Social Security, public assistance, pensions, or retirement benefits).

1. Was your total HOUSEHOLD income in [INSERT LAST YEAR]?
2. Below $40,000
3. $40,000 or more
4. Don’t know

Respondents were prompted to answer question 11 only if they selected option “a” for question 10.

1. And was your total HOUSEHOLD income in [INSERT LAST YEAR]?
2. Below $20,000
3. $20,000 or more
4. Don’t know

Respondents were prompted to answer question 12 only if they selected option “a” for question 11.

1. Which one of the following includes your total HOUSEHOLD income in [INSERT LAST YEAR] before taxes?
2. Less than $5,000
3. $5,000 to $9,999
4. $10,000 to $14,999
5. $15,000 to $19,999
6. Don’t know

Respondents were prompted to answer qu^4,26^estion 13 only if they selected option “b” for question 11.

1. Which one of the following includes your total HOUSEHOLD income in [INSERT LAST YEAR] before taxes?
2. $20,000 to $24,999
3. $25,000 to $29,999
4. $30,000 to $34,999
5. $35,000 to $39,999
6. Don’t know

Respondents were prompted to answer question 14 only if they selected option “b” for question 10.

1. Was your total HOUSEHOLD income in [INSERT LAST YEAR]?
2. Below $85,000
3. $85,000 or more
4. Don’t know

Respondents were prompted to answer question 15 only if they selected option “a” for question 14.

1. Which one of the following includes your total HOUSEHOLD income in [INSERT LAST YEAR] before taxes?
   1. $40,000 to $49,999
   2. $50,000 to $59,999
   3. $60,000 to $74,999
   4. $75,000 to $84,999
   5. Don’t know

Respondents were prompted to answer question 16 only if they selected option “b” for question 14.

1. Which one of the following includes your total HOUSEHOLD income in [INSERT LAST YEAR] before taxes?
   1. $85,000 to $99,999
   2. $100,000 to $124,999
   3. $125,000 to $149,999
   4. $150,000 to $174,999
   5. $175,000 to $199,999
   6. $200,000 or more
   7. Don’t know
2. What is the highest level of school you have completed?
   1. No formal education
   2. 1st, 2nd, 3rd, or 4th grade
   3. 5th or 6th grade
   4. 7th or 8th grade
   5. 9th grade
   6. 10th grade
   7. 11th grade
   8. 12th grade NO DIPLOMA
   9. High School Graduate – high school diploma or the equivalent (GED)
   10. Some college, no degree
   11. Associate degree
   12. Bachelor’s degree
   13. Master’s degree
   14. Professional or Doctorate degree

**C. Sensitivity Analysis:** **Effects of Civic Engagement on Rates of Moderate Psychological Distress Among U.S. Adults in November 2020**

|  | **Model 1^a^** | **Model 2^b^** | **Model 3^c^** | **Model 4^d^** |
| --- | --- | --- | --- | --- |
|  | **n=1000** | **n=999** | **n=1000** | **n=999** |
| **Type of Organization** | Linear regression coefficients (standard error) | | | |
| Unknown | 45.5 (7.8) | 41.0 (7.4) | 46.5 (7.8) | 41.8 (7.5) |
| Business or Professional Organizations | 46.2 (7.8) | 45.1 (7.9) | 46.2 (7.8) | 45.6 (7.9) |
| Community, Arts & Rec Organizations | 48.2 (5.1) | 47.8 (5.4) | 48.0 (5.1) | 47.8 (5.4) |
| Identity-based Organizations | 52.1 (3.8) | 51.3 (3.8) | 52.2 (3.8) | 51.5 (3.8) |
| Political Organizations | 64.0 (5.6) | 61.8* (5.7) | 61.0** (5.6) | 57.9*** (6.2) |
| Religious Organizations | 35.6 (3.6) | 38.1 (3.6) | 35.3 (3.6) | 37.6 (3.7) |
| Social Services Organizations | 42.9 (4.6) | 41.9 (4.7) | 43.0 (4.5) | 41.8 (4.7) |
| **Type of Engagement/Nature of Interaction** |  |  |  |  |
| **Average hours interacting with the organization each week** |  |  |  |  |
| None | 37.2 (4.3) |  | 36.4 (4.4) |  |
| 1-2 | 46.6 (2.6) |  | 46.7* (2.6) |  |
| 2-5 | 44.6 (4.0) |  | 43.7 (3.9) |  |
| 5+ | 51.7* (4.4) |  | 52.0 (4.4) |  |
| **Frequency of interactions with people at the organization** |  |  |  |  |
| Never |  | 49.4 (4.8) |  | 48.1 (5.0) |
| Occasionally |  | 46.8 (2.6) |  | 46.8 (2.6) |
| Regularly |  | 41.3 (3.1) |  | 41.2 (3.1) |
| **Political organization x hours interacting each week** |  |  |  |  |
| None |  |  | 70.8 (9.4) |  |
| 1-2 |  |  | 62.6 (7.9) |  |
| 2-5 |  |  | 35.3**^1^ (15.1) |  |
| 5+ |  |  | 81.3 (12.7) |  |
| **Political organization x frequency of interactions with people** |  |  |  |  |
| Never |  |  |  | 77.3 (7.4) |
| Occasionally |  |  |  | 60.3 (9.0) |
| Regularly |  |  |  | 45.1*^1^ (12.1) |
| **Demographic Controls** |  |  |  |  |
| **Gender** |  |  |  |  |
| Male | 40.3 (2.7) | 39.8 (2.8) | 40.1 (2.7) | 39.7 (2.8) |
| Female | 50.4** (2.4) | 50.5** (2.4) | 50.5** (2.4) | 50.6** (2.4) |
| **Race/Ethnicity** |  |  |  |  |
| White, non-Hispanic | 50.1 (2.2) | 50.0 (2.2) | 50.1 (2.1) | 50.0 (2.2) |
| Black, non-Hispanic | 38.5* (5.0) | 38.7* (5.1) | 38.1* (5.0) | 39.0 (5.1) |
| Other, non-Hispanic | 30.0* (7.2) | 30.8* (7.2) | 29.8* (7.0) | 30.6* (7.2) |
| Hispanic | 41.5 (5.0) | 40.3 (5.2) | 42.0 (5.1) | 40.5 (5.2) |
| **Age** |  |  |  |  |
| 18-34 | 64.4 (4.1) | 64.1 (4.2) | 64.1 (4.1) | 64.1 (4.2) |
| 35-49 | 50.6* (3.6) | 50.7* (3.6) | 50.2* (3.6) | 50.3* (3.6) |
| 50-64 | 35.2*** (3.3) | 34.8*** (3.4) | 35.6*** (3.3) | 34.9*** (3.4) |
| 65+ | 31.3*** (3.6) | 31.8*** (3.7) | 31.7*** (3.6) | 32.1*** (3.7) |
| **Household Income** |  |  |  |  |
| <$35,000 | 43.6 (3.6) | 42.9 (3.6) | 43.7 (3.6) | 43.0 (3.6) |
| $35,000-$74,999 | 47.4 (3.0) | 46.8 (3.0) | 47.0 (3.0) | 46.6 (3.1) |
| $75,000+ | 45.4 (3.3) | 46.1 (3.3) | 45.6 (3.3) | 46.2 (3.3) |
| **Education Level** |  |  |  |  |
| High school diploma or less | 46.2 (4.2) | 45.4 (4.2) | 46.1 (4.2) | 45.5 (4.2) |
| Some college or more | 45.3 (1.8) | 45.5 (1.9) | 45.3 (1.8) | 45.4 (1.9) |
| **Essential worker status** |  |  |  |  |
| Not essential worker or unknown | 46.4 (2.1) | 46.3 (2.2) | 46.4 (2.1) | 46.3 (2.1) |
| Essential worker | 43.1 (3.6) | 42.9 (3.7) | 43.2 (3.6) | 42.8 (3.7) |
| **Married/Partnered status** |  |  |  |  |
| Nor partnered | 50.0 (3.1) | 49.6 (3.2) | 50.3 (3.2) | 49.7 (3.2) |
| Partnered | 42.7 (2.3) | 42.7 (2.4) | 42.5 (2.3) | 42.6 (2.4) |

**Notes:** *p≤0.05, **p≤0.01, ***p≤0.001 statistically significant from reference category. Standard errors are included in parentheses. Table reflects predicted probabilities of moderate psychological distress calculated from logistic regression models. Psychological distress was measured using the Kessler-6 scale, dichotomized to reflect moderate psychological distress (defined as a score above 4). Associational characteristics were collected in Wave 1. Sex, race/ethnicity, age, household income, education, and married/partnered status were collected as part of the baseline NORC Amerispeak panel. Psychological distress and the essential worker variable were evaluated in Wave 3.

^1^ Indicates use of a one-tailed significance test, based on our hypothesis that more hours spent with an association and more interpersonal interactions would be associated with lower rates of psychological distress.

^a^ Model 1 examines the main effects of hours spent with the association and type of association.

^b^ Model 2 examines the main effects of frequency of interactions with people in the association and type of association.

^c^ Model 3 examines the main effects of the interaction between hours spent with the association and belonging to a political association.

^d^ Model 4 examines the main effects of the interaction between frequency of interactions with people and belonging to a political association.
